# Rapid Evaluation of the Pozzolanic Activity of Bayer Red Mud by a Polymerization Degree Method: Correlations with Alkali Dissolution of (Si+Al) and Strength

**DOI:** 10.3390/ma14195546

**Published:** 2021-09-24

**Authors:** Yaguang Wang, Xiaoming Liu, Zhiqing Xie, Huimin Wang, Wei Zhang, Yang Xue

**Affiliations:** 1 State Key Laboratory of Advanced Metallurgy, University of Science and Technology Beijing, Beijing 100083, China; wangyg@xs.ustb.edu.cn; 2 School of Metallurgical and Ecological Engineering, University of Science and Technology Beijing, Beijing 100083, China; xiezhiqing8023@163.com (Z.X.); 41802169@xs.ustb.edu.cn (H.W.); b20190124@xs.ustb.edu.cn (W.Z.); cdxueyang@163.com (Y.X.)

**Keywords:** Bayer red mud, pozzolanic activity, evaluation method, polymerization degree, dissolution of (Si+Al)

## Abstract

A large amount of Bayer process red mud is discharged in the process of alumina production, which has caused significant pollution in the environment. The pozzolanic activity of Bayer red mud as a supplementary cementitious material is a research hotspot. In this work, a new method for Fourier-transform infrared spectrometry is used to determine the polymerization degree of Bayer red mud in order to evaluate its pozzolanic activity. Based on the results of the dissolution concentration of (Si+Al), strength index and polymerization degree of Bayer red mud, the relationships between different evaluation methods were analyzed, and the relevant calculation formulas of pozzolanic activity were obtained. The results showed that different evaluation methods can reflect the variation law of pozzolanic activity in Bayer red mud. The polymerization degree of Bayer red mud had a good linear relationship with the pozzolanic activity index obtained by the strength index and dissolution concentration of (Si+Al), respectively. The polymerization degree was negatively correlated with pozzolanic activity index and dissolution concentration of (Si+Al), and the correlation coefficients were greater than 0.85. Therefore, this method was found to be effective and hence can be used as a rapid and simple test for pozzolanic activity evaluation of Bayer red mud.

## 1. Introduction

Bayer red mud (RM) is a kind of strong alkaline solid waste discharged in the process of alumina production; about 1–2 tons of RM will be discharged per ton of alumina production [1,2,3]. At present, the annual discharge of RM has exceeded 100 million tons in China, which is mainly disposed of by damming, and the comprehensive utilization rate is less than 4% [3]; this stacked RM has caused serious pollution to the surrounding environment. In order to solve the problem of RM pollution, the comprehensive utilization of RM has become a focus for researchers.

Meanwhile, increases in infrastructure worldwide have led to a substantial increase in the demand for cement. However, cement production not only consumes a large amount of coal, limestone, iron ore, clay and other resources, but also discharges a large amount of CO_2_, which seriously pollutes the environment. The green sustainable development of cement has been a concern of international scholars, and research focus has mainly been on energy conservation, emission reduction and environmental protection, in order to meet the needs of carbon neutrality. Industrial solid wastes, such as fly ash, and blast furnace and steel slag, have been widely used as supplementary cementitious materials in the field of cement and concrete [4,5,6].

RM can be used as a supplementary cementitious material for cement and concrete, which can greatly improve the resource utilization rate of RM. However, the current utilization rate of RM in cementitious materials is low because of its high alkalinity and low pozzolanic activity [3]. The pozzolanic activity of industrial solid waste is a hot topic in current research [7]. Some methods for evaluating pozzolanic reactivity were proposed based on the principle of interactions between calcium hydroxide and industrial solid waste with potential pozzolanic activity. Moreover, some simple, rapid and quantitative methods have been used to evaluate the pozzolanic activity of industrial solid waste; these methods include the chemical composition of industrial solid waste, crystallinity of minerals, and strength and electrochemical performance [8,9,10,11,12]. In addition, although strength evaluation can comprehensively and intuitively reflect the overall pozzolanic activity of materials, the required cycle is usually long. The dissolution evaluation must select appropriate conditions to separate the active components from the inert components. However, there are many influencing factors in the implementation of this method, such as the type, concentration, and dissolution time and temperature of alkali solutions, which will lead to large errors in the experimental results. In recent years, some scholars have studied new methods for testing the pozzolanic activity of materials. Martín et al. [13] measured the pozzolanic activity of hollow glass microspheres through the improved Chapelle test and compared the compressive strength of hardened cement paste samples, and were able to obtain better results. Basto et al. [14]. studied the pozzolanic activity of sewage sludge ash via a conductivity method. The results show that there is a good correlation between mortar compressive strength tests and conductivity tests. Hasani et al. [15]. proposed a new method to simply compare pozzolanic activities by using the molecular dynamics simulation method. The adsorption process of water molecules on the surface of pozzolanic minerals was simulated, and the water/surface interaction energy was used as a criterion for investigating mineral activity. However, the physical and chemical properties of different kinds of industrial solid wastes are greatly different, which leads to difficulties in establishing a model that accurately evaluates the pozzolanic activity of industrial solid wastes. It is worth noting that there are many factors affecting the pozzolanic activity of RM. Therefore, it is of great importance to find a quick and effective method for determining the pozzolanic activity of RM.

In this work, the evaluation of pozzolanic activity of RM by polymerization degree was proposed. The pozzolanic activity of calcined RM at different temperatures was evaluated by the methods of strength, dissolution of (Si+Al) and polymerization degree. The polymerization degree of RM was characterized by FTIR. Correlations between polymerization degree and other evaluation methods of pozzolanic activity were established, providing a theoretical reference for the application of RM in the field of cement and concrete.

## 2. Materials and Methods

RM was produced by the China Aluminum Co., Ltd. Shanxi Branch, Hejin, China. Cement (42.5) was purchased from the Tangshan Jidong Cement Co., Ltd, Tangshan, China. Standard sand was purchased from a factory in Henan, China. Sodium hydroxide was purchased from Sinopharm Chemical Reagent Co., Ltd, Beijing, China. Deionized water was made in the laboratory. An XRF was used to analyze the main chemical constituents of RM and cement. The main chemical constituents of RM and cement are shown in Table 1. The main chemical constituents of RM are Fe_2_O_3_, Al_2_O_3_, SiO_2_, Na_2_O and a small amount of CaO.

First, 200 g of RM was weighed and put into a muffle furnace (△S1200, Zhengzhou Ansheng Scientific Instrument Co., Ltd, Zhengzhou, China). The muffle furnace was heated to a set temperature at a rate of 10 °C/min (the set temperatures were 100 °C, 200 °C, 300 °C, 400 °C, 500 °C, 600 °C, 700 °C, 800 °C, 900 °C and 1000 °C, respectively). After reaching the set temperature, the muffle furnace was kept warm for 2 h. After calcination, the RM was cooled to room temperature, and the RMs showing differing pozzolanic activities were obtained; the RMs were named RMn (*n* = 100–1000) for different calcination temperatures. Finally, the pozzolanic activity of RM was evaluated according to different experimental methods.

Strength method: The pozzolanic activity index of RM was calculated according to the Chinese standard GB/T 2847–2005 (pozzolanic materials used for cement production) [16]. The masses of RMs with different pozzolanic activities, the cement and the standard sand were 135 g, 315 g and 1350 g, respectively; these were stirred and mixed according to a water–cement ratio of 0.5, and then injected into an abrasive tool to vibrate and form. The prepared mortar sample was put into the curing box (YH-40B, Hebei Ruiheng test instrument factory, Cangzhou, China), the formwork removed after curing for 24 h, and curing continued until 28 days. The curing temperature was 20 ± 1 °C, and the curing humidity was 95%. The compressive strength of the sample was tested with the press (DYE-300, Beijing hengying Technology Co., Ltd, Beijing, China), with 6 test samples for each sample. The pozzolanic activity index (Kα) of RM was expressed by the compressive strength percentage of sample A (RM and cement system) at 28 days and sample B (cement system) at 28 days:

Kα = (compressive strength of sample A)/(compressive strength of sample B) × 100%(1)

Alkali dissolution of (Si+Al) method: One gram of RMs with differing pozzolanic activities were taken and put into a 100 mL plastic bottle with 1 mol/L of NaOH solution. After sealing, it was put into a curing room at 20 °C for 7 days before filtering. The filtrate was sealed and stored in a plastic bottle. The contents of Si^4+^ and Al^3+^ in the filtrate were tested by ICP–OES (ICPOES730, Agilent Technologies, Palo Alto, CA, USA) [17]. It is worth noting that the more silicon and aluminum ions that were dissolved, the higher the pozzolanic activity of the RM.

Polymerization degree method: Origin software (Origin 2018, OriginLab, Northampton, MA, USA) was used to split and fit the peak area of Si(Al)Q^n^ in the range of 800–1200 cm^−1^ on the FTIR spectrum. Zhang et al. [18] proposed the concept of the relative bridging oxygen bond (RBO) to evaluate the polymerization degree of [SiO_4_]. The polymerization degree was calculated by the following formula:

(2) $$\mathrm{RBO}=\frac{1}{4}(1\times \frac{Q^{1}}{\Sigma Q^{n}}+2\times \frac{Q^{2}}{\Sigma Q^{n}}+3\times \frac{Q^{3}}{\Sigma Q^{n}}+4\times \frac{Q^{4}}{\Sigma Q^{n}})=\frac{1}{4}\times \frac{\Sigma n\times Q^{n}}{\Sigma Q^{n}}$$

The main chemical compositions of RM and cement were analyzed by X-ray fluorescence spectrometry (XRF; xrf-1700, Shimadzu, Shimadzu enterprise management (China) Co., Ltd, Shanghai, China). The phase composition of RM and cementitious materials was measured by an X-ray diffractometer (XRD; D/max Rb, Rigaku Corporation, Tokyo, Japan). The experimental conditions were 40 kV, 100 mA, Cu Target and scanning speed of 4 °/min. The molecular bonds of the samples were tested by Fourier-transform infrared spectrometry (Nicolet IS10, Thermo Nicolet Corporation, Madison, GA, USA). The test conditions were as follows: sample and KBr were put into a mortar, ground and mixed evenly (1 mg of sample and 100 mg of KBr). After that, it was pressed into a transparent sheet of a specified size, and the measured wavenumber range was 400–4000 cm^−1^.

## 3. Results and Discussion

### 3.1. XRD of RMs with Different Pozzolanic Activities

**Figure 1:**
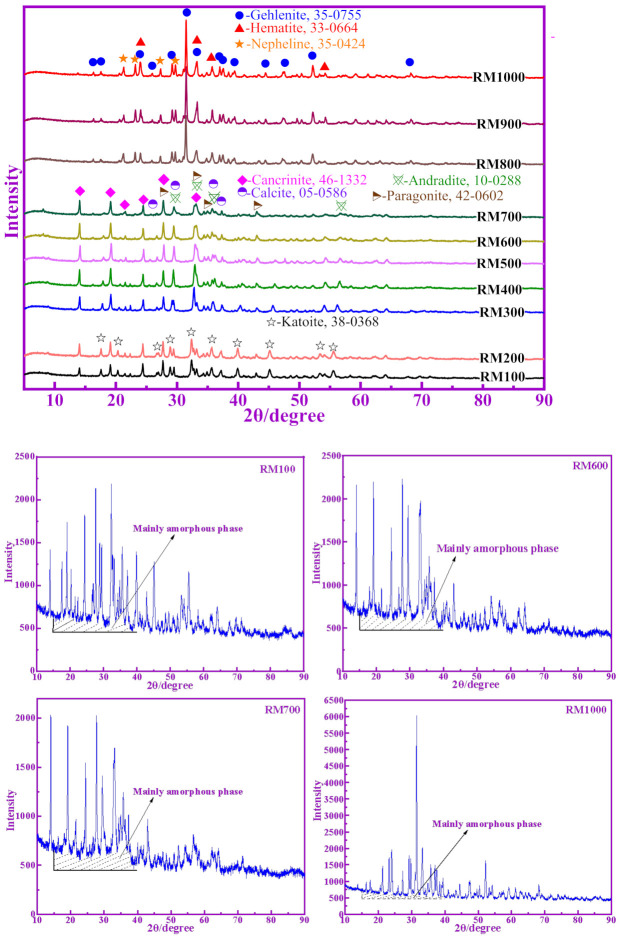
XRD patterns of calcined RM at different temperatures.

The phase composition has an important influence on the pozzolanic activity of RM. Previous studies have shown that the amorphous phase is the most active phase in supplementary cementitious materials, such as GGBFS and fly ash [3]. The XRD patterns of RM calcined at different temperatures, as shown in Figure 1. It can be seen from Figure 1 (RM100) that the diffraction peaks of different phases in RM are clear and sharp, which indicates that there are few amorphous phase substances in RM, and that the crystallization degree of each phase is high. The phase analysis of RM shows that its main phase composition is composed of katoite, cancrinite, calcite, paragonite and diaspore. It is worth noting that the XRD patterns of RM100 and RM200 are similar, the XRD patterns of RM300–700 are similar, and the XRD patterns of RM800–1000 are similar. This shows that the phase composition of RM changes gradually with increases in temperature.

In the XRD patterns of RM calcined at ≥300 °C, the diffraction peaks for diaspore gradually disappear, which indicates that the diaspore has been decomposed—but no crystal peaks of Al_2_O_3_ have been found, indicating that the diaspore has decomposed to produce active Al_2_O_3_, which is difficult to detect in XRD [19]. At 300–700 °C, katoite in RM begins to decompose hydroxyl and to transform into cancrinite. When the temperature rises to 700 °C, the diffraction peak of katonite disappears completely. At the same time, the peak of calcite also decreased gradually, indicating that it also decomposed.

The phase composition of RM800–1000 is mainly composed of gehlenite, hamatite and nepheline. It is worth noting that these are newly formed substances. This shows that the original substances in RM have undergone phase transformation. After 800 °C, the higher the temperature, the stronger the peak of crystalline phase in RM, indicating that the content of crystalline minerals in RM is relatively increased.

In addition, it can be seen from Figure 1 showing RM100, RM600, RM700 and RM1000, that the content of the amorphous phase in calcined RM at different temperatures is different. The content of the amorphous phase in RM700 is the highest and that in RM1000 is the lowest. This means that calcined RM has different pozzolanic activities at different temperatures.

The phase transformation reactions of RM during calcination at different temperatures are as follows:

200–700 °C:

AlO(OH) → Al_2_O_3_ + H_2_O (3)

Aluminosilicate minerals → Active silicon and aluminum(4)

CaCO_3_ → CaO + CO_2_ (5)

800–1000 °C:

CaCO_3_ → CaO + CO_2_ (6)

Active silicon and aluminum → Aluminosilicate minerals(7)

CaO + Al_2_O_3_ + SiO_2_ → Gehlenite (8)

### 3.2. Compressive Strength Evaluation of Pozzolanic Activity

The compressive strength of RM and cement mortar is used as an index to evaluate pozzolanic activity. The compressive strength of mortar is a comprehensive index reflecting its structure; it can not only reflect the secondary reaction of RM in the hardening process, but can also reflect its filling water reduction effects. Therefore, the compressive strength test can comprehensively reflect the role of pozzolanic materials in the whole system.

**Figure 2:**
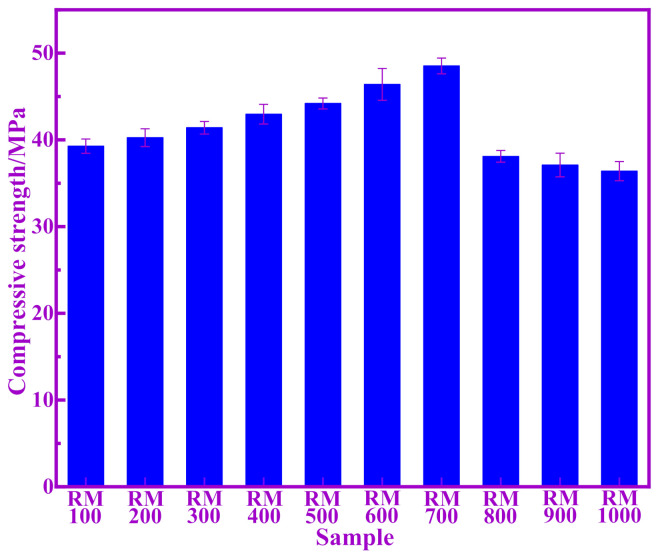
The compressive strength of samples at different temperatures.

Figure 2 shows the compressive strength of cement paste of calcined RM at different temperatures at 28 days. It can be seen from Figure 2 that the compressive strength of calcined RM mortar at different temperatures first increases and then decreases with temperature. The compressive strength of RM700 is the highest, reaching 48.52 MPa. Table 2 shows the pozzolanic activity index of calcined RM at different temperatures (he compressive strength of the standard cement mortar used is 62.80 MPa at 28 days). It can be seen from Table 2 that the change law of the pozzolanic activity index of calcined RM at different temperatures is consistent with that of its compressive strength. This indicates that RM700 has the highest activity of Si and Al, which is consistent with the area of amorphous peak in XRD. The compressive strength of RM1000 decreased significantly, and its compressive strength at 28 days was lower than that of RM100. The compressive strength and pozzolanic activity index of RM1000 were the lowest, indicating that the content of active Si and Al were the lowest, basically not participating in the hydration reaction, and mainly playing a filling role. The above results show that the pozzolanic activity of RM changes distinctly at different calcination temperatures, and that the pozzolanic activity of RM700 is the highest.

### 3.3. Pozzolanic Activity Evaluation for Dissolution of Silicon and Aluminum

**Figure 3:**
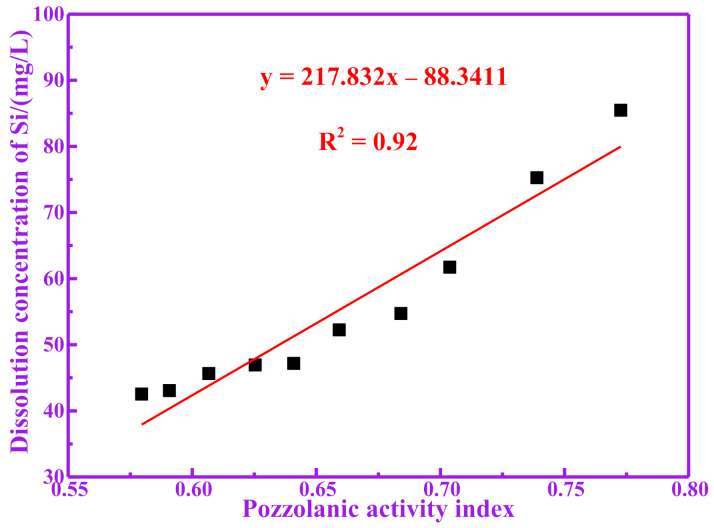
Correlation between dissolution concentration of Si and pozzolanic activity index.

**Figure 4:**
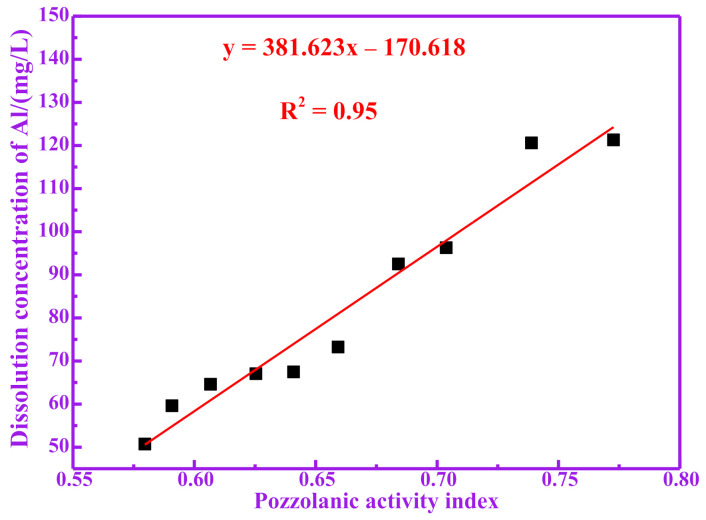
Correlation between dissolution concentration of Al and pozzolanic activity index.

**Figure 5:**
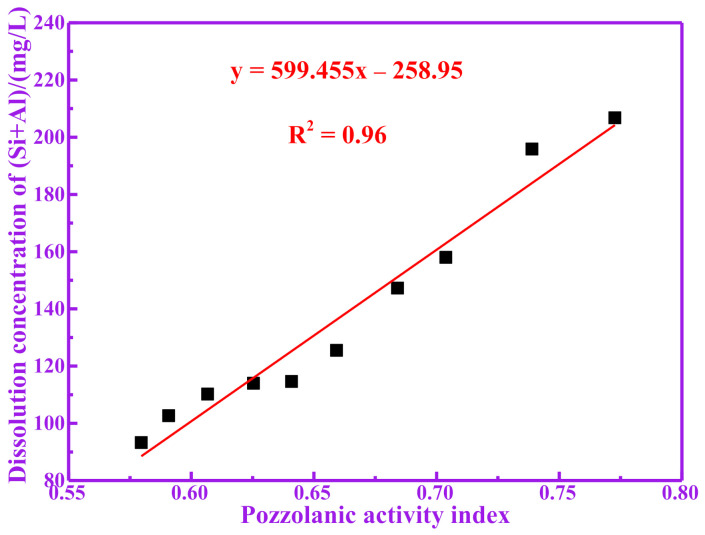
Correlation between dissolution concentration of (Si+Al) and pozzolanic activity index.

Active silicon and aluminum produced by the decomposition of pozzolanic materials are considered to be the main sources of pozzolanic activity [20,21]. Silicon and aluminum components that can be dissolved under alkaline conditions may participate in the hydration reaction of cement. Therefore, the higher the content of silicon and aluminum that can be dissolved in the RM, the higher the degree of the possible hydration reaction of the RM, and the more hydration products it produces [22]. For studying the dissolution amounts of silicon and aluminum in the active components of calcined RM in alkali solution, it is a more direct evaluation method to take the dissolution amount of silicon and aluminum in the solution as the index for evaluating the pozzolanic activity. Table 3 shows the dissolved concentrations of Si, Al and (Si+Al) of RM at different temperatures in NaOH solution. It can be seen from Table 3 that the dissolution of active silicon and aluminum increases first and then decreases with the calcination temperature. The dissolution amounts of silicon and aluminum in RM700 are 85.47 mg/L and 121.30 mg/L, respectively. The dissolution amounts of silicon and aluminum in RM1000 are 42.53 mg/L and 50.71 mg/L, respectively. It is worth noting that the change trend for the dissolution amount of silicon and aluminum with temperature is consistent with that of the pozzolanic activity index. Therefore, the relationship between the pozzolanic activity index of RM and its dissolution amount of silicon and aluminum has been studied. The relationship between the pozzolanic activity index of RM and its dissolution concentration of Si is shown in Figure 3. The relationship between the pozzolanic activity index of RM and its dissolution concentration of Al is shown in Figure 4. The relationship between the pozzolanic activity index of RM and its dissolution concentration of (Si+Al) is shown in Figure 5. It can be seen from Figure 3, Figure 4 and Figure 5 that there is a good correlation between the dissolution concentration of Si, Al and (Si+Al) in RM and its pozzolanic activity index, and there is a positive correlation (linear tendency) between them; the higher the dissolution concentration of Si, Al and (Si+Al) is, the higher the pozzolanic activity index of RM is. The linear fitting R^2^ between pozzolanic activity of RM and dissolution concentration of Si, Al and (Si+Al) is 0.92, 0.95 and 0.96, respectively. It is worth noting that the linear fitting R^2^ between the dissolution concentration of (Si+Al) and the pozzolanic activity index is the largest, indicating that there is a good correlation between the pozzolanic activity of RM and the dissolution concentration of (Si+Al). Therefore, the dissolution amount of silicon and aluminum in RM is closely related to its pozzolanic activity.

### 3.4. Relationship between Polymerization Degree and Pozzolanic Activity for RM

**Figure 6:**
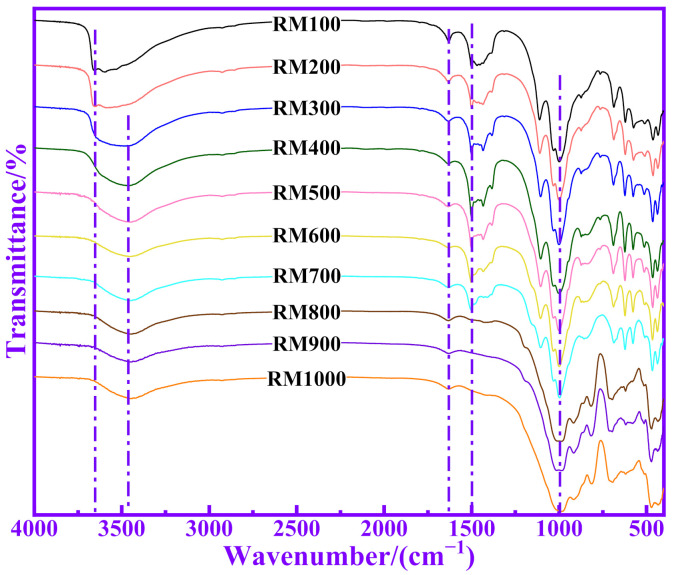
The FTIR spectrum of calcined RM at different temperatures.

The chemical structural changes of RM calcined at different temperatures were analyzed by FTIR. Figure 6 shows the FTIR spectra of RM calcined at different temperatures. The absorption peak is 3638 cm^−1^, which corresponds to the stretching vibration of free –OH [3]. The absorption peak at 3452 cm^−1^ corresponds to the stretching vibration of associating –OH. With increases in temperature, the free –OH in RM is gradually transformed into bound –OH, which indicates that there is a certain amount of hydrogen bonding in its molecular structure. The absorption peaks of 1635 cm^−1^ and 1431 cm^−1^ correspond to the stretching vibration and antisymmetric stretching vibration of C–O, respectively. It can be seen from Figure 6 that the carbonate in RM gradually decomposes with increases in temperature. The absorption peak in the range of 800–1200 cm^−1^ is an asymmetric tensile vibration of Si–O–Si or Si–O–Al connected with a tetrahedron of [SiO_4_] or [AlO_4_]^−^ [22]. The peak at 1092 cm^−1^ corresponds to the tensile vibration of O–Si–(Si). The peak at 998 cm^−1^ is caused by the tensile vibration of Si–O (Al). The peak at 871 cm^−1^ corresponds to the tensile vibration of Si–O–. The absorption peak in the range of 600–800 cm^−1^ is symmetrical to stretching vibrations between Si–O–(Si, Al) in tetrahedrons of [SiO_4_] or [AlO_4_]^−^ [23]. It can be seen from Figure 6 that the position and area of peaks in 800–1200 cm^−1^ changes with temperature, which indicates that the bond between Si, Al and O in RM is broken or combined. The absorption peak of 582 cm^−1^ corresponds to the rings of Si–O and Al–O. The peaks in the range of 400–500 cm^−1^ correspond to the bending vibration of Si–O–Si (Al).

The essence of pozzolanic materials participating in the hydration reaction of cement is the process of [Si(Al)O_4_] tetrahedrons changing from their polymerization state, to their isolated state, back to their polymerization state; the increase in pozzolanic activity is caused by the depolymerization of polymerized [Si(Al)O_4_] tetrahedrons (i.e., the fracture of the Si–O–Si(Al) bond). Generally, the smaller the polymerization degree of the silicon aluminum network is, the higher its pozzolanic activity is.

**Figure 7:**
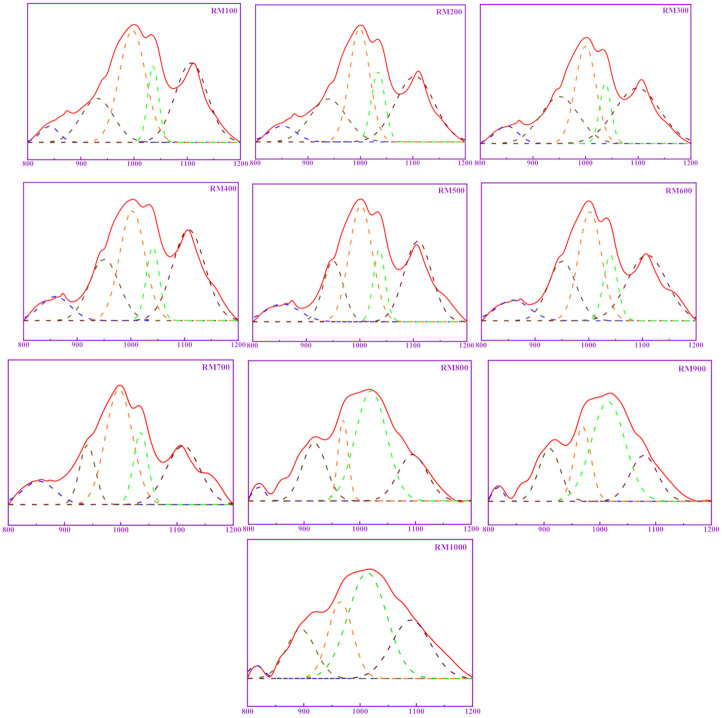
The peaks in the range of 800–1200 cm^−1^ of calcined RM at different temperatures.

According to the number of coordination bridge oxygens around a Si, it can be divided into SiQ^0^, SiQ^1^, SiQ^2^, SiQ^3^ and SiQ^4,^ where n in SiQ^n^ represents the number of coordination bridge oxygens around Si. The fracture of the Si–O–Si bond in [SiO_4_] can cause changes in the Si coordination structure around bridge oxygens, that is, from SiQ^n^ to SiQ^n−1^. On the contrary, if Si–O and Si–O polymerize to form a Si–O–Si bond, the Si coordination structure around the bridge oxygens will change from SiQ^n^ to SiQ^n+1^. It is worth noting that the Al in the system can enter the [SiO_4_] during the polymerization process to replace the Si and form [AlO_4_]^−^, but it does not affect the change in the number of bridge oxygen bonds. Therefore, the change in bridge oxygen number can be used to reflect the relative degree of polymerization or depolymerization in the system. Zhang et al. [17]. proposed the concept of RBO to evaluate the polymerization degree of [SiO_4_]. Therefore, here the authors used the polymerization degree to reflect the pozzolanic activity of calcined RM at different temperatures. In FTIR spectra, the characteristic peaks of SiQ^0^, SiQ^1^, SiQ^2^, SiQ^3^, and SiQ^4^ are about 850 cm^−1^, 950 cm^−1^, 1000 cm^−1^, 1050 cm^−1^, and 1100 cm^−1^, respectively. Origin software was used to separate and fit peaks between 800–1200 cm^−1^, whereas the peak area and RBO were calculated. Figure 7 shows the peaks in the range of 800–1200 cm^−1^ of the FTIR spectra of calcined RM at different temperatures. Table 4 shows the relevant parameters of the peaks of calcined RM at different temperatures. It can be seen from Table 4 that the polymerization degree of RM first decreases and then increases with the increase in temperature. The high polymerization degree indicates that the silicon and aluminum component has stable chemical properties. The low polymerization degree indicates that the Si–O–Si (Al) bond in the RM is destroyed, which will increase the active silicon and aluminum in the RM.

**Figure 8:**
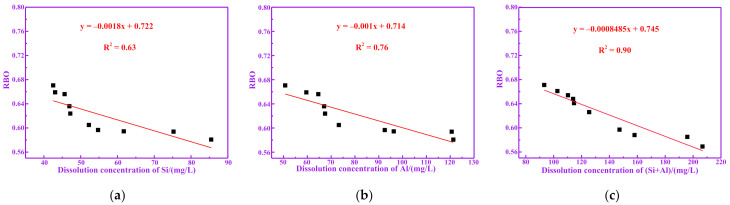
Correlation between dissolution concentration of (Si+Al) and RBO. (**a**) Si; (**b**) Al; (**c**) (Si+Al).

Figure 8a–c show the relationship between RBO and dissolution concentration of Si, Al and (Si+Al) of calcined RM at different temperatures, respectively. It can be seen from Figure 8a–c that RBO tends to be linear to the dissolution concentration of Si, Al and (Si+Al), respectively. Therefore, the lower the RBO of RM is, the higher the dissolution concentration of Si, Al and (Si+Al) is. Therefore, the decrease in RBO promoted an increase of the dissolution concentration of Si, Al and (Si+Al) in RM. According to the results of the XRD, this behavior is due to the conversion of crystalline phase substances in the RM into active Si and Al at 700 °C, resulting in the increase in the dissolution concentration of Si, Al and (Si+Al).

**Figure 9:**
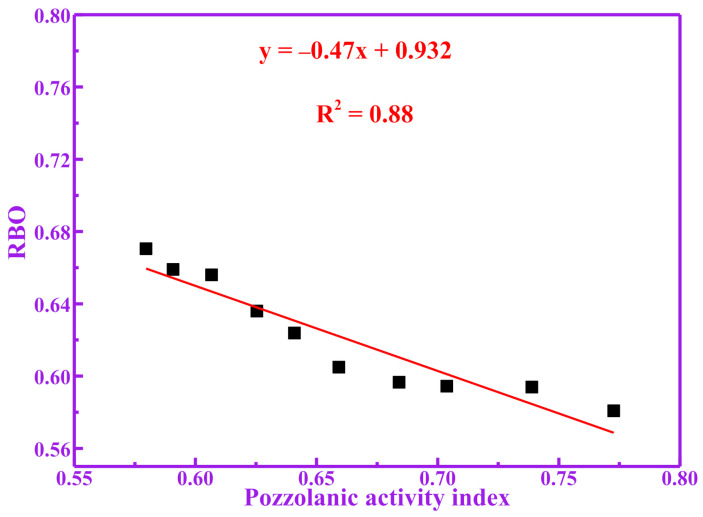
Correlation between RBO and pozzolanic activity index.

Figure 9 shows the relationship between RBO and pozzolanic activity of calcined RM at different temperatures. It can be seen from Figure 9 that there is a significant correlation between the pozzolanic activity of RM and its RBO. Therefore, the trend of this relationship is that the smaller the RBO of RM, the higher the pozzolanic activity that is obtained by RM. Therefore, compared with the compressive strength and dissolution of silicon and aluminum, the polymerization degree evaluation method of pozzolanic activity is simple and fast. At the same time, the method also obtains similar results. The most important thing is that the proposed method is adequate to verify the pozzolanic activity of RM.

## 4. Conclusions

In this work, a new method to evaluate the pozzolanic activity of RM by its polymerization degree was proposed. The relationships between compressive strength, dissolution concentration of (Si+Al) and polymerization degree method with pozzolanic activity of RM were studied, respectively. A correlation between polymerization degree method, compressive strength and dissolution concentration of (Si+Al) was established. The main findings of this work are as follows:

The pozzolanic activity of RM can be calculated quantitatively by polymerization degree method and compressive strength and dissolution concentration of (Si+Al). The polymerization degree of RM had a good linear relationship with the pozzolanic activity index, obtained by the strength index and dissolution concentration of (Si+Al), respectively.

The polymerization degree of RM decreases with increases in pozzolanic activity, which is negatively correlated. The compressive strength and dissolution concentration of (Si+Al) are positively correlated with the pozzolanic activity of RM. The fitting equation between the polymerization degree and the pozzolanic activity index of RM was y = −0.47x + 0.932, where R^2^ was 0.88. The fitting equation between the polymerization degree and the dissolution concentration of (Si+Al) of RM was y = −0.0008485x + 0.745, where R^2^ was 0.90.

Compared to the compressive strength, with a test cycle of 28 days, and the dissolution of (Si+Al), with a test cycle of 7 days, the polymerization degree method has the advantages of a short test cycle, convenience and low cost. It is related to the structure of silicon and aluminum in RM and is not easily affected by the test environment. Therefore, it is a fast, simple and reliable method to evaluate the pozzolanic activity of RM by polymerization degree method.

Through research on the relationship between the polymerization degree of RM and its pozzolanic activity, a linear relationship between them was found. However, this work only studied one industrial solid waste—RM. From the results of this work, the polymerization degree method may be applicable to the pozzolanic activity evaluation of many kinds of industrial solid wastes. In the future, the authors will try to apply this method to other solid wastes other than RM. In conclusion, this work provides a reference for the study of fast and reliable evaluation methods of pozzolanic activity of industrial solid waste in the future.

## Figures and Tables

**Table 1:** The chemical constituents of RM and cement (wt.%).

Oxides	SiO_2_	Al_2_O_3_	Fe_2_O_3_	CaO	MgO	Na_2_O	SO_3_	TiO_2_	LOI
RM	22.71	22.96	26.57	2.17	0.13	11.08	1.01	1.72	11.19
Cement	18.60	3.81	3.25	64.86	2.90	0.24	0.45	0.31	4.14

**Table 2:** The pozzolanic activity index of calcined RM at different temperatures.

Sample	RM100	RM200	RM300	RM400	RM500	RM600	RM700	RM800	RM900	RM1000
Kα	62.54%	64.09%	65.92%	68.41%	70.38%	73.89%	77.27%	60.67%	59.08%	57.96%

**Table 3:** The dissolution concentration of Si and Al in calcined RM at different temperatures (mg/L).

Sample	Dissolution Concentration of Si	Dissolution Concentration of Al	Dissolution Concentration of (Si+Al)
RM100	46.93	67.04	113.97
RM200	47.18	67.44	114.62
RM300	52.24	73.24	125.48
RM400	54.73	92.51	147.24
RM500	61.72	96.29	158.01
RM600	75.25	120.60	195.85
RM700	85.47	121.30	206.77
RM800	45.63	64.60	110.23
RM900	43.06	59.60	102.66
RM1000	42.53	50.71	93.24

**Table 4:** The relevant parameters of the peaks of calcined RM at different temperatures.

Sample	Relative Content/%					RBO	R^2^
	SiQ^0^	SiQ^1^	SiQ^2^	SiQ^3^	SiQ^4^		
RM100	3.20	17.21	35.12	10.95	33.52	0.6360	0.995
RM200	4.41	20.11	30.04	12.42	33.02	0.6238	0.995
RM300	5.43	24.20	27.24	9.26	33.87	0.6049	0.995
RM400	7.85	19.87	29.98	10.38	31.92	0.5966	0.995
RM500	8.07	15.98	35.40	11.22	29.33	0.5944	0.994
RM600	8.28	19.21	30.30	11.07	31.14	0.5939	0.994
RM700	9.74	12.05	39.65	13.29	25.27	0.5808	0.987
RM800	2.13	19.39	11.39	48.13	18.96	0.6560	0.992
RM900	2.07	15.83	17.03	46.56	18.51	0.6590	0.995
RM1000	1.93	14.75	19.23	41.39	22.7	0.6705	0.988

## Data Availability

Data sharing is not applicable to this article.
